# How Sensitive Are Conventional MEG Functional Connectivity Metrics With Sliding Windows to Detect Genuine Fluctuations in Dynamic Functional Connectivity?

**DOI:** 10.3389/fnins.2019.00797

**Published:** 2019-08-02

**Authors:** Lucrezia Liuzzi, Andrew J. Quinn, George C. O’Neill, Mark W. Woolrich, Matthew J. Brookes, Arjan Hillebrand, Prejaas Tewarie

**Affiliations:** ^1^Sir Peter Mansfield Imaging Centre, School of Physics and Astronomy, University of Nottingham, Nottingham, United Kingdom; ^2^Oxford Centre for Human Brain Activity, University of Oxford, Warneford Hospital, Oxford, United Kingdom; ^3^Oxford Centre for Functional MRI of the Brain, University of Oxford, John Radcliffe Hospital, Oxford, United Kingdom; ^4^Department of Clinical Neurophysiology and MEG Center, Amsterdam Neuroscience, Amsterdam UMC, Vrije Universiteit Amsterdam, Amsterdam, Netherlands

**Keywords:** dynamic functional connectivity, magnetoencephalography, multivariate autoregressive models, sliding window, neural mass model

## Abstract

Despite advances in the field of dynamic connectivity, fixed sliding window approaches for the detection of fluctuations in functional connectivity are still widely used. The use of conventional connectivity metrics in conjunction with a fixed sliding window comes with the arbitrariness of the chosen window lengths. In this paper we use multivariate autoregressive and neural mass models with *a priori* defined ground truths to systematically analyze the sensitivity of conventional metrics in combination with different window lengths to detect genuine fluctuations in connectivity for various underlying state durations. Metrics of interest are the coherence, imaginary coherence, phase lag index, phase locking value and the amplitude envelope correlation. We performed analysis for two nodes and at the network level. We demonstrate that these metrics show indeed higher variability for genuine temporal fluctuations in connectivity compared to a static connectivity state superimposed by noise. Overall, the error of the connectivity estimates themselves decreases for longer state durations (order of seconds), while correlations of the connectivity fluctuations with the ground truth was higher for longer state durations. In general, metrics, in combination with a sliding window, perform poorly for very short state durations. Increasing the SNR of the system only leads to a moderate improvement. In addition, at the network level, only longer window widths were sufficient to detect plausible resting state networks that matched the underlying ground truth, especially for the phase locking value, amplitude envelope correlation and coherence. The length of these longer window widths did not necessarily correspond to the underlying state durations. For short window widths resting state network connectivity patterns could not be retrieved. We conclude that fixed sliding window approaches for connectivity can detect modulations of connectivity, but mostly if the underlying dynamics operate on moderate to slow timescales. In practice, this can be a drawback, as state durations can vary significantly in empirical data.

## Introduction

Large-scale functional interactions in the brain are assumed to be mirrored by statistical dependencies between time evolving activity of neuronal populations, which can be quantified by magnetoencephalography (MEG), electroencephalography (EEG) and functional (f)MRI. The spatiotemporal patterns of these functional interactions (functional connectivity) are still commonly treated in a “static connectivity” sense, i.e., functional connectivity estimates are obtained by collapsing connectivity over windows. There is ample evidence that neuronal interactions appear in spatial clusters or subnetworks that form and dissolute over time (Breakspear et al., 2004; O’Neill et al., 2015, 2017a; de Pasquale et al., 2016). By collapsing connectivity over time windows, we potentially miss crucial information about the temporal evolution of these clusters. Furthermore, recent electrophysiological studies have demonstrated that taking into account the temporal domain of connectivity may give insight into abnormal brain function in neurological diseases (Stam et al., 2005; Carbo et al., 2017; Kim et al., 2017; Watanabe and Rees, 2017). Therefore, there is a need to include the dynamics of functional interactions into our analysis. In addition, given the superior temporal resolution of MEG and EEG to fMRI, these modalities could offer a means to analyze the dynamics of functional interactions in more detail.

A conventional approach to tackle dynamic functional connectivity is to use fixed sliding window approaches, i.e., a window of fixed length is moved in time, using an overlapping or non-overlapping approach, and “static” connectivity is estimated within every fixed window (O’Neill et al., 2017b). This approach comes with the arbitrariness of the window length. Recent approaches have demonstrated that dynamic connectivity can be well-described at a variety of time-scales, therefore a fixed window length may not be appropriate to capture the underlying fluctuations in connectivity (Baker et al., 2014; Vidaurre et al., 2016, 2018; Brookes et al., 2018; Tewarie et al., 2018; Tronarp et al., 2018). In other words, there may be a mismatch between the temporal scale of the underlying fluctuations and the predefined fixed window length, potentially leading to erroneous estimates of connectivity. Nevertheless, studies have used the sliding window approach during cognitive tasks and also in neurological disease (O’Neill et al., 2017b). Of special interest in these studies was the variability of connectivity, which was shown to relate better to outcome measures, such as cognitive decline after neurosurgical intervention (Carbo et al., 2017), than static connectivity information. However, it is known from the fMRI literature that variability of connectivity does not necessarily imply that the underlying system is non-stationary or dynamic (Hindriks et al., 2016; Laumann et al., 2016). Furthermore, most of the popular functional connectivity metrics have mainly been systematically evaluated in the static connectivity sense (David et al., 2004; Wang et al., 2014; Colclough et al., 2016; Dimitriadis et al., 2018). Therefore, there is a need to understand the strengths and limitations of commonly used metrics of functional connectivity when used with a fixed sliding window approach.

In the current work, we systematically analyze the sensitivity and specificity of the fixed sliding window approach in conjunction with commonly used connectivity metrics to detect genuine fluctuations in connectivity. We opted to include connectivity metrics based on the two important intrinsic modes of connectivity, i.e., phase- and amplitude based methods (Siegel et al., 2012). In order to perform this analysis, we require a system with a known ground truth regarding the strength and duration of the time varying connections. Given the lack of this information in empirical MEG data, we employ two models: a parameterized neural mass model (NMM) (Jansen and Rit, 1995) and a parameterized multivariate autoregressive model (MAR) (Neumaier and Schneider, 2001) that both provide *a priori* defined ground truths. Simulations are performed for a two node system and a large scale network. The former allows us to test the performance of the metrics without any external nuisance factors, while the latter allows us to test the performance of the metrics in a more realistic scenario. We first test in a two node system the null hypothesis that the observed variability of connectivity estimates can merely be understood in terms of an underlying static system superimposed by noise. Secondly, we analyze the correlation between, and error in, the connectivity estimates compared to the underlying ground truth for different state durations and window lengths. In other words, are the different metrics sensitive to the same or different time-scales of dynamic connectivity? Are results from connectivity metrics only valid within a limited temporal range? We then test the ability of metrics to capture the underlying fluctuations in states for different signal-to-noise ratios (SNR), since shorter window lengths also come with the limitation of lower SNR. We finally evaluate in large scale network simulations if connectivity metrics can retrieve switching between *a priori* defined resting state networks over time and whether the spatial connectivity patterns can be retrieved using non-negative tensor factorization. This is performed in a system without and with linear mixing (in order to model signal leakage).

## Materials And Methods

### Multivariate Autoregressive Model

**FIGURE 1:**
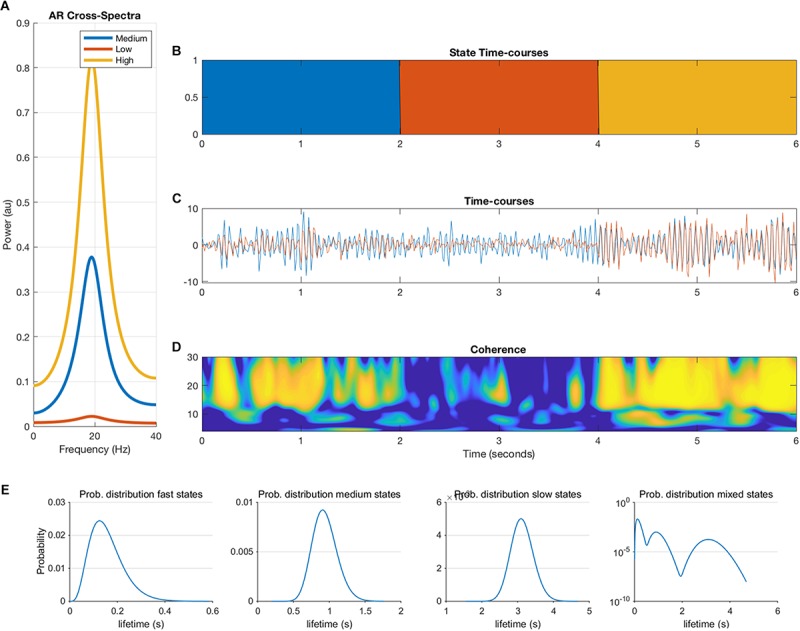
Overview of the multivariate autoregressive model. The cross-spectra **(A)**, state timecourses **(B)**, the two nodal timecourses **(C)**, the coherence between the two nodes **(D)** are illustrated. Panel **(E)** shows the ground-truth probability distributions of the state lifetimes for fast, medium, slow and mixed states, which are input to the model (note that the *y*-axis of the mixed states is in logarithmic scale).

The multivariate autoregressive (MAR) model describes data observations of a system at time *t* (*X*_t_) as a linear mapping (*A*) of *p* past observations

(1) $$X_{t}=\sum_{\tau =1}^{p}A_{\tau }\,X_{t-\tau }+e,$$

where *e* is additive Gaussian noise, and *p* = *2*. This autoregressive model is an infinite impulse response or all-pole linear filter whose frequency content is determined by the roots of the polynomial *A*. These roots can be estimated by an eigendecomposition of the companion form of the parameter matrix (Neumaier and Schneider, 2001). Each mode of the decomposition is defined by a resonant frequency described by the eigenvalue and a projection into the channels in *X* described by the eigenvectors. Each state in the simulations is defined by a 2-node MAR model with a single resonance defined by its eigen-parameters with a frequency, magnitude and weight in each node. The weight into node 1 is held fixed whilst the weight in node 2 varies between 0 and 0.9 to create a phase locked coupling of different strengths. These eigen-parameters are then transformed back into the temporal parameter matrix *A*. New realizations of the system can then be generated by filtering white noise with the pre-defined *A* matrix. Each parameter matrix A produces time-series with a fixed phase lag between nodes 1 and 2 at a given coupling strength. Note that this will inherently favor phase based metrics over amplitude metrics in our simulations. Three parameter settings were created, corresponding to three states with low, medium and high coupling strength. Duration of the states, i.e., duration of ground truth connectivity was determined by a Gamma distribution. Figure 1 shows an example of the output of the MAR model. We generated 300 s of data for each simulation and iteration and all data were band pass filtered into the beta band (15–25 Hz).

### Parameterized Neural Mass Model

**FIGURE 2:**
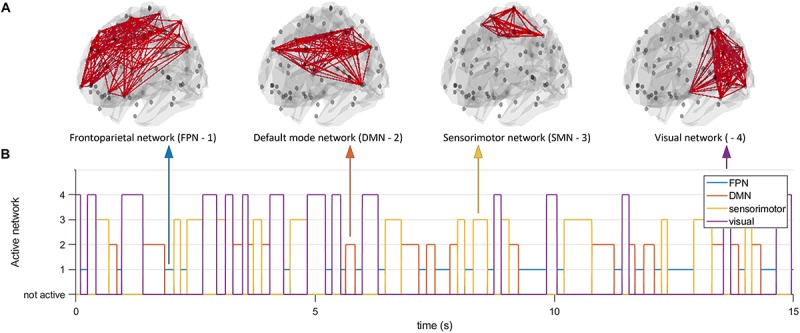
Time evolving activity of resting state subnetworks. We simulated a network of 78 neural mass units, which were connected using structural connectivities that resembled one of four resting state networks (default mode network, frontoparietal network, sensorimotor network, and a visual network **(A)**). At every moment in time only the connections underlying one resting state network were active **(B)**, all other nodes were active but not connected. Note that the number on the *y*-axis does not indicate the level of connectivity, but merely the network that was active at any given point in time. At every moment in time we estimated functional connectivity within an active resting state subnetwork and outside the active resting state subnetwork.

Since simulations are characterized by model specific features of the data that will inherently influence connectivity estimates, we opted to also use a different model to generate data to assess generalizability of results across models for the two node system. At the same time, this gave us the opportunity to analyze a large scale network with conduction delays. We employed the well-known Jansen and Rit NMM (Jansen and Rit, 1995). Every unit in the NMM consisted of an excitatory population, an inhibitory population, and a pyramidal population whose activity mimicked the MEG/EEG signal. Simulations were fed with a Gaussian white-noise process and equations were solved using a stochastic Heun’s integration scheme with a time step of 1 × 10^–4^ (Tewarie et al., 2019b). Parameters were the same as used in Grimbert and Faugeras (2006). We generated data for the working point of the model well beyond a Hopf bifurcation (i.e., in the limit cycle regime), with the rationale that no unpredictable switching could occur between the limit cycle regime and the linear regime due to noise. Hence, in this way a controllable system for the analysis of dynamic functional connectivity was achieved. We first connected two nodes using a structural coupling parameter *k*. For the two node system, coupling *k* was parameterized as a time-series, instead of a constant as is typically done. Duration of the states for *k*, and hence the duration of ground truth connectivity was again determined by a Gamma distribution. The parameterization of *k* was realized in the same way as described above for the MAR model (with fast, medium, slow and mixed states). We generated 300 s of data for each simulation and iteration and all data were band pass filtered into the alpha band (8–13 Hz). In the second part of the simulations, we simulated activity for *N* = 78 nodes, corresponding to the cortical regions in the automated anatomical atlas (AAL) (Tzourio-Mazoyer et al., 2002). This number of nodes also roughly matches the potential number of independent sources for the relatively low spatial dimensionality of MEG/EEG data (Farahibozorg et al., 2018). To boost neurobiological realism, we included distance dependent conduction delays in our simulations (Cabral et al., 2014; Tewarie et al., 2019b). To this end, the Euclidean distances between centroids of the parcels in the AAL atlas was used and divided by conduction velocity (*v* = 10 m/s). For the large scale network simulations, we kept *k* constant throughout the simulations, but fed the simulations with a structural connectivity tensor (instead of a structural connectivity matrix). This structural connectivity tensor had dimensions *N × N × T*, where refers *T* to the duration of the simulation. At every time point the structural connectivity tensor was characterized by a resting state subnetwork connectivity state. The resting state networks of interest were the default mode network (DMN), sensorimotor network (SMN), the frontoparietal networks (FPN), and the visual network. The duration of the states was again determined by a Gamma distribution (with fast, medium, slow and mixed states). For every time point only one resting state network was active (see Figure 2), with the other nodes being active, but not connected. The resting state networks were obtained from independent component analysis of fMRI data (at the voxel level) projected onto the AAL atlas in standard MNI space [data obtained from Tewarie et al. (2016)].

### MEG Connectivity Metrics

We employ methods that capture information from two modes of connectivity, i.e., amplitude and phase based metrics. We apply connectivity metrics to beta band (MAR) and alpha band (NMM) filtered timecourses for both amplitude and phase based metrics (i.e., amplitude envelope correlation, phase locking value, phase lag index, coherence and imaginary coherence). The implementation of all the metrics is exactly the same as in Liuzzi et al. (2017).

1. *Amplitude envelope correlation (AEC)* (Brookes et al., 2011; Hipp et al., 2012): The amplitude envelopes are extracted using the Hilbert transform of band pass filtered data and the Pearson correlation is computed between the amplitude envelopes of two pair of regions.

2. *Coherence (COH) and Imaginary Coherence (iCO)* (Nolte et al., 2004)*:* Coherency is computed on band pass filtered timecourses by evaluating

(2) $$C\,\left(f\right)=\frac{S_{x\,y}\,\left(f\right)}{\sqrt{S_{x\,x}\,\left(f\right)\,S_{y\,y}\,\left(f\right)}},$$

where *S*_xy_ denotes the cross spectral density for two timecourses when *x*≠*y* or the auto spectral densities when *x=y*. The coherence was computed from the absolute value of coherency and by averaging over the frequency band of interest. Imaginary Coherence can also be extracted from Eq. 2 by simply calculating the imaginary part of coherency *C(f)* thus removing zero lag phase relationships.

3. *Phase Locking Value (PLV)* (Lachaux et al., 1999) characterizes a stable phase relationship between two timecourses within a predefined window. The instantaneous phases are derived using the Hilbert transform and the difference between the instantaneous phases *i* and *j* at time *t* denoted as Δφ_*ij*_(*t*). Phase locking is subsequently defined as

(3) $$P\,L\,V=\left|\left\langle e^{i\,\Delta \,\varphi_{i\,j}\,\left(t\right)}\right\rangle \right|$$

4. *Phase Lag index (PLI)* (Stam et al., 2007): The PLI uses similar phase information as the PLV but discards zero-lag phase differences. Unlike the PLV, it merely quantifies the asymmetry of the phase difference distribution

(4) $$P\,L\,I=\left|\left\langle \text{sign}\,\left(\text{ℑ}\,\left[e^{i\,\Delta \,\varphi_{i\,j}\,\left(t\right)}\right]\right)\right\rangle \right|.$$

### Extracting Time Varying Networks Using Non-negative Tensor Factorization

We extracted time evolving subnetworks using non-negative tensor factorization. This method can be considered as a higher order principal component analysis and decomposes a third order tensor into a set of basis vectors (Bro, 1997; Gauvin et al., 2014)

(5) $$T=\sum_{l=1}^{L}a_{l}\times b_{l}\times c_{l}$$

Here × corresponds to the outer product, and *a*_*l*_, *b*_*l*_ and *c*_*l*_ correspond to basis vectors of component *l* and have dimensions *N* (network size) and *t* (number of time points). In other words, the outer product of *a*_*l*_ and *b*_*l*_ reflects the connectivity patterns characterized by the time-series *c*_*l*_. The factorization of tensor *T* is found by solving the optimization problem $m\,i\,n_{A,B,C}\,∥{\text{T-T}}_{A,B,C}^{\prime }∥$, with the constraints of orthogonality of the first two basis vectors (*a*_*k*_, *b*_*k*_), and non-negativity of the last vectors *c*_*k*_ (where $T_{A,B,C}^{\prime }$ is the approximation of *T*_*A,B,C*_). The N-way toolbox (version 1.8) in Matlab was used for this analysis (Andersson and Bro, 2000). We set *L* = *4*, given the four resting state networks that were used in the simulations.

### Analysis Steps

In order to analyze the sensitivity of our connectivity metrics to genuine fluctuations in connectivity, we follow a step-by-step approach. Analysis is divided into two parts: (1) simulations for a two node system and (2) simulations for a large scale network.

#### Two Node System Analysis

##### Dynamic vs. static connectivity

We first simulated two conditions: (1) two timecourses with an underlying static connectivity between them, superimposed by noise, and (2) two timecourses with underlying fluctuations in connectivity for three different mean state durations (125 ms, 1 s, 3 s, mixed state durations based on the latter three mean state durations). These different state lifetimes and mean state durations were obtained by tuning the scale and shape parameters for a Gamma distribution. Then, for the different metrics we computed the connectivity using a sliding window approach with windows 50% overlapping in time, resulting in a distribution of connectivity values for the entire simulation. We calculated four summary statistics from the distribution: (1) standard deviation, (2) skewness, (3) kurtosis, (4) excursions from the median. The latter three statistics were selected as resulting connectivity distributions, especially for the “dynamic connectivity case” can be non-Gaussian. The metric “excursions from the median” has extensively been described in Zalesky et al. (2014) and Hindriks et al. (2016), and captures the length and height of all excursions from the median. The rationale is that, the longer and larger the excursions from the median, the greater the evidence for non-stationarity of connectivity. We *a priori* expected all four metrics to be larger in the dynamic connectivity case than in the static connectivity case. A range of window lengths were chosen for this analysis. For every window we ran twenty iterations. This was done for both the static connectivity case as well as for the dynamic connectivity case. The null hypothesis (i.e., observed variability of connectivity estimates can be fully accounted for by an underlying static system superimposed by noise) can be rejected if the summary statistic in the dynamic system exceeds the summary statistic of the static system. For every window length we computed the Mann–Whitney test to reject the null hypotheses.

##### Detecting genuine fluctuations in connectivity

We evaluated for the different metrics the mean error of the connectivity estimate. The error was defined as the mean absolute difference between underlying ground truth and connectivity estimates. This was again done for different mean state durations and for a range of window lengths. Similarly, we computed the Pearson correlation coefficient between the underlying ground truth and the connectivity fluctuations obtained with the different metrics. For both outcome measures, prior to calculation, we interpolated the connectivity estimates using a cubic spline interpolation. Interpolation was necessary since there was a dimension mismatch between the underlying ground truth connectivity timecourses and the connectivity estimates obtained with the five metrics [i.e., the latter were based on one estimate for every window, whereas in that window the underlying connectivity was fully sampled as the data timecourse (i.e., the ground truth)]. We used non-parametric Friedman tests to test the effect of window length on the correlations and mean error (test for repeated measures). This was done in order to analyze whether a change in the distributions of the correlations or mean absolute error was significantly different for subsequent window lengths.

##### The effect of SNR

We evaluated the effect of different SNR for different mean state durations on the ability of connectivity metrics to detect the underlying ground-truth connectivity. Again the correlation between the estimates and the underlying ground truths was evaluated.

#### Network Analysis

##### Detecting temporal fluctuations of resting state subnetworks

We evaluated whether the connectivity metrics are sensitive to detect switching of resting state subnetworks over time. Similar as for the two node system analysis, we temporally interpolated the estimated functional connectivity data since there was a dimension mismatch with the dimension of the structural connectivity tensor (time interval of a window is reduced to a single estimate for functional connectivity). Again note that there was only one resting state network active at every time point (DMN, SMN, FPN or visual), and the duration of the states (fast, medium, slow, mixed) determined the switching between four different resting state subnetworks. For every time point *t* in the simulation, we tested whether the strength of within resting state network connectivity exceeded the magnitude of connectivity outside the resting state network at every point in time. This was tested for the a priori defined connections belonging to a resting state subnetwork.

##### Detecting temporal fluctuations of resting state subnetworks with linear mixing

The former analysis was repeated in a more realistic scenario. Source localization for empirical MEG/EEG data usually involves signal leakage, which manifests itself as a zero-time lag linear summation of underlying signals. Here, instead of a forward projection in our simulations from the network source nodes to sensors and an inverse projection to the sources (Chella et al., 2019), we opted to implement linear mixing to model such signal leakage (Lobier et al., 2014). Linear mixing was induced by adding for a time-series of a node *i* the weighted activity of all other nodes using a linear combination, ${\tilde{x}}_{i}=x_{i}+\overset{N}{\underset{j,j\neq i}{\text{∑}}}\frac{1}{{\text{d}}_{\text{ij}}}\,{\text{x}}_{\text{j}}$, where the weights were chosen as the inverse Euclidean distance between two nodes *i* and *j*. Lastly, prior to the calculation of the connectivity-metrics that are sensitive to signal leakage (coherence, amplitude envelope correlation and phase locking value), we used a symmetric multivariate leakage correction method to reduce the effects of signal leakage (Colclough et al., 2015). For the windows and metrics that did show significant differences in connectivity for the *a priori* defined connections within subnetworks and outside subnetworks, we also tested whether time varying subnetworks could be retrieved from the data itself using non-negative tensor factorization.

## Results

### Two Node System: Estimates of Static vs. Dynamic Connectivity

**FIGURE 3:**
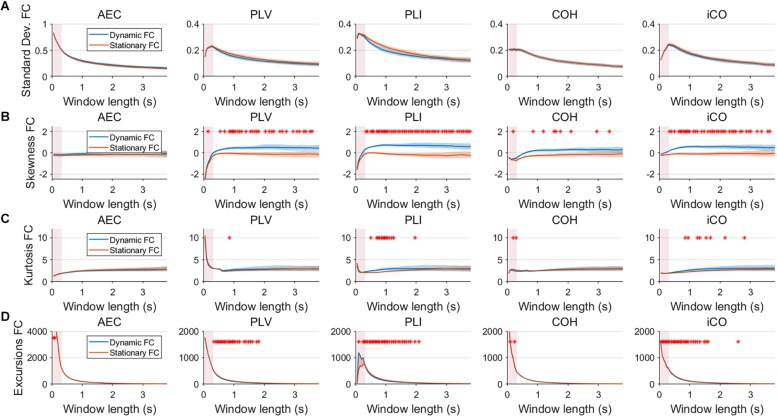
Two node MAR model summary statistics for connectivity in a static vs. fast dynamic underlying system (SNR = 1). Panel **(A)** shows the standard deviation of connectivity across the number of windows for the static and dynamic underlying system (pink rectangles correspond to the underlying ground-truth of the range of the state durations). Panel **(B)** shows the skewness of the connectivity distributions. Panel **(C)** shows the kurtosis of the connectivity distributions and panel **(D)** the excursions from the median. Shaded areas correspond to the range across realizations/iterations. A red cross corresponds to a significant difference in distribution between a summary statistic for static and dynamic connectivity (Mann–Whitney test *p* < 0.01) for the window length of interest.

**FIGURE 4:**
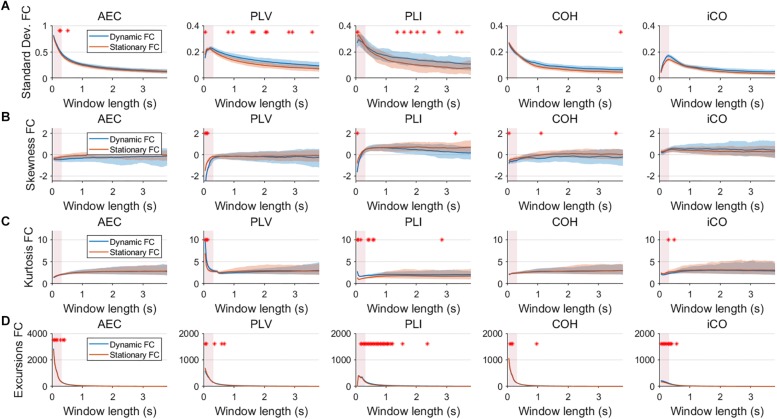
Two node NMM model summary statistics for connectivity in a static vs. fast dynamic underlying system (SNR = 1). Panel **(A)** shows the standard deviation of connectivity across the number of windows for the static and dynamic underlying system (pink rectangles correspond to the underlying ground-truth of the range of the state durations). Panel **(B)** shows the skewness of the connectivity distributions. Panel **(C)** shows the kurtosis of the connectivity distributions and panel **(D)** the excursions from the median. Shaded areas correspond to the range across realizations/iterations. A red cross in each panel corresponds to a significant difference in distribution between a summary statistic for static and dynamic connectivity (Mann–Whitney test *p* < 0.01) for the window length of interest.

**FIGURE 5:**
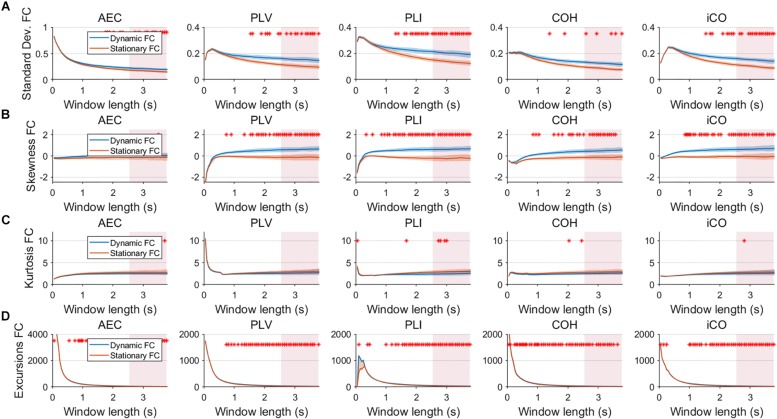
Two node MAR model summary statistics in connectivity in a static vs. slow dynamic underlying system (SNR = 1). Panel **(A)** shows the standard deviation of connectivity across the number of windows for the static and dynamic underlying system (pink rectangles correspond to the underlying ground-truth of the range of the state durations). Panel **(B)** shows the skewness of the connectivity distributions. Panel **(C)** shows the kurtosis of the connectivity distributions and panel **(D)** the excursions from the median. Shaded areas correspond to the range across realizations/iterations. A red cross in each panel corresponds to a significant difference in distribution between a summary statistic for static and dynamic connectivity (Mann–Whitney test *p* < 0.01) for the window length of interest.

**FIGURE 6:**
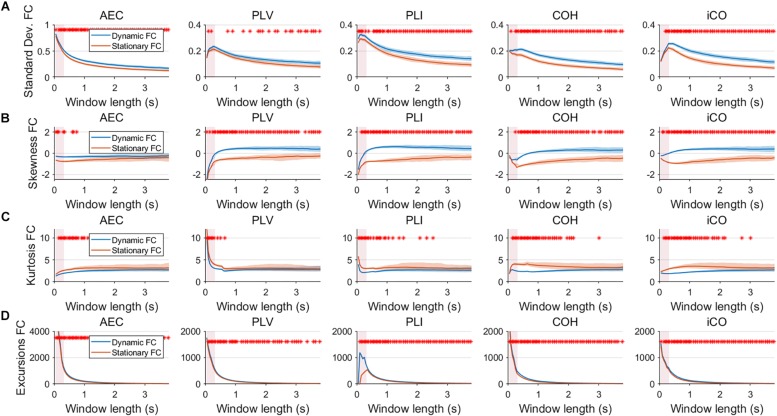
Two node MAR model summary statistics for connectivity in a static vs. fast dynamic underlying system (SNR = 3). Panel **(A)** shows the standard deviation of connectivity across the number of windows for the static and dynamic underlying system (pink rectangles correspond to the underlying ground-truth of the range of the state durations). Panel **(B)** shows the skewness of the connectivity distributions. Panel **(C)** shows the kurtosis of the connectivity distributions and panel **(D)** the excursions from the median. Shaded areas correspond to the range across realizations/iterations. A red cross in each panel corresponds to a significant difference in distribution between a summary statistic for static and dynamic connectivity (Mann–Whitney test *p* < 0.01) for the window length of interest.

Fluctuations for dynamic connectivity in our simulations were characterized by fast, medium, slow and mixed states. Results are shown for the MAR and NMM models and for an SNR of 1 (Figures 3–5) and for an SNR of 3 (Figure 6) for the MAR model for the fast states. Figure 3A shows the variability (standard deviation) of connectivity estimates for the fast dynamic and the static system as a function of window length. We *a priori* expected higher variability for the fast dynamic case, since in its underlying timecourse of connectivity there was a frequent switch between three coupling values (low, medium, high connectivity), whereas for the static case there was constant coupling during the whole simulation. For all metrics, we could see that for an SNR of 1 connectivity estimates from a genuine dynamic connectivity timecourse showed the same variability as connectivity estimates based on the static connectivity timecourse (Mann–Whitney test for all windows *p* > 0.01). For both the static and dynamic case, variability generally decreased as a function of the window length. A difference in skewness was observed between static connectivity (Figure 3B) and dynamic connectivity with higher skewness for dynamic connectivity for all measures but the AEC, but usually in window lengths that did not match the underlying state durations. Note that the positive skewness for the dynamic case for most metrics indicates that the connectivity distribution was non-Gaussian. Kurtosis and excursions from the median were significantly larger for the dynamic connectivity than for the static connectivity (Figures 3C,D), although for both, mostly not for the window widths that matched the underlying state durations. Although note that for the excursions from the median this was also significant for the correct window lengths. Simulations based on the NMM for an SNR of 1 and fast state durations showed similar results for most metrics and summary statistics (Figure 4). Again, excursions from the median was significantly higher for dynamic connectivity, especially for the window widths that matched the underlying state durations. Similar curves were also obtained for medium (Supplementary Figure S1), mixed (Supplementary Figure S2) and slow states (Figure 5) for the MAR model and NMM model (Supplementary Figures S3–S5), with the difference that especially for slow states there was a clear divergence in variability, skewness, kurtosis and excursions from the median between static and dynamic connectivity (Figure 5) for several window lengths, including the longer window lengths that matched the underlying state durations. Note that curves for fast states, medium and slow states have very similar shapes for both MAR and NMM, indicating that the form of these curves are largely determined by window length rather than state durations.

Having said this, increasing the SNR resulted in a better disentanglement of dynamic vs. static connectivity with our conventional metrics. Figure 6 shows the results for fast states for an SNR of 3. In contrast to results for an SNR of 1 (Figure 3), the range of the standard deviation for static and dynamic connectivity start to diverge for most metrics for even short window lengths (Figure 6A). A clear divergence between static and dynamic connectivity was also observed for skewness (Figure 6B), as well as for kurtosis (Figure 6C) and excursions from the median. Results for medium, slow and mixed states for an SNR of 3 showed similar disentanglement of dynamic vs. static connectivity (see Supplementary Figures S6–S8).

### Two Node System: Identifying Genuine Fluctuations in Dynamic Connectivity

**FIGURE 7:**
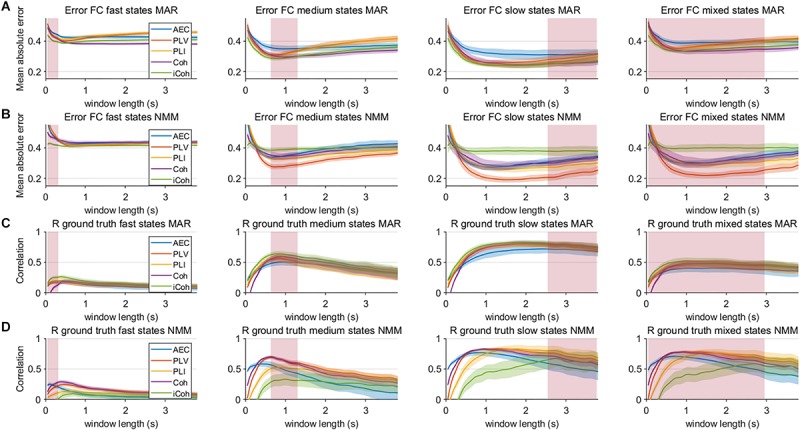
Mean error and correlation between connectivity estimates and ground truth for the two node MAR and NMM models. Panels **(A,B)** show the mean absolute error of the estimated connectivity relative to the underlying ground truth for different state durations and window lengths, and for the MAR and NMM model, respectively. Panels **(C,D)** show the correlation between interpolated connectivity estimates and ground truth connectivity timecourse for different window lengths for the MAR and NMM model, respectively. Pink rectangles show the range of the underlying state durations. Shaded areas correspond to the range.

Results in the previous section show that especially for sufficient long window lengths, metrics could identify higher and genuine variability for the dynamic underlying system. However, this only indicates that metrics have some sensitivity to pick up differences between coupling and uncoupling, it does not indicate whether the correct duration of the state lifetimes could be captured. In order to address this, we computed the mean error and the correlation coefficient between ground truth connectivity timecourse and (interpolated) estimated connectivity timecourse. Figures 7A,B displays the mean error for the connectivity estimates for different metrics and state durations and for the different models (pink rectangle shows the range of true underlying state durations). This shows that the error increases for shorter state durations and for shorter window lengths for both the MAR and NMM simulations. For medium and slow state durations there seems to occur a plateau or minimum for the window lengths that roughly match the duration of the states. For fast states, there is no clear difference between the metrics in the window lengths that matches the underlying ground truth connectivity modulations. However, for slow states the error of the AEC is larger than for the other metrics for the MAR model, while for the NMM model the error of iCO is larger than for other metrics. There was a significant effect of window length for all metrics and state durations on the mean absolute error (see Table 1). Similar results can be observed for the correlation with the ground truth, i.e., the shorter the state durations, the lower the correlation of the connectivity estimates with the ground truth (Figures 7C,D). For short state durations, there is a poor correlation with the ground truth for all the metrics and for both NMM and MAR simulations. Again, there are maxima at, or around, (e.g., for short state durations) the window lengths that match the underlying state durations. There is no clear difference in performance for the different metrics in the MAR simulations, apart from the observation that the AEC underperforms for longer state durations. Whereas for the NMM simulations the iCO seem to perform worse for longer and mixed state durations. Again, there was a significant effect of window length for all metrics and state durations on the correlation with the ground truth (see Table 1).

**TABLE 1:** Non-parametric Friedman statistics for all correlation and mean absolute error for all metrics.

	**AEC**		**PLV**		**PLI**		**COH**		**iCO**	
	**χ^2^(79)**	***p***	**χ^2^(79)**	***p***	**χ^2^(79)**	***p***	**χ^2^(79)**	***p***	**χ^2^(79)**	***p***
**MAR model**										
Fast-states R	213	<0.001	560	<0.001	523	<0.001	234	<0.001	429	<0.001
Fast-states Error	441	<0.001	383	<0.001	413	<0.001	453	<0.001	510	<0.001
Medium-states R	327	<0.001	576	<0.001	575	<0.001	504	<0.001	574	<0.001
Medium-states Error	483	<0.001	530	<0.001	529	<0.001	550	<0.001	557	<0.001
Slow-states R	299	<0.001	444	<0.001	471	<0.001	417	<0.001	462	<0.001
Slow-states Error	451	<0.001	510	<0.001	489	<0.001	515	<0.001	514	<0.001
Mixed-states R	223	<0.001	525	<0.001	523	<0.001	367	<0.001	505	<0.001
Mixed-states Error	261	<0.001	323	<0.001	313	<0.001	369	<0.001	373	<0.001
**NMM model**										
Fast-states R	472	<0.001	595	<0.001	278	<0.001	644	<0.001	259	<0.001
Fast-states Error	307	<0.001	419	<0.001	407	<0.001	186	<0.001	211	<0.001
Medium-states R	642	<0.001	714	<0.001	525	<0.001	719	<0.001	351	<0.001
Medium-states Error	631	<0.001	664	<0.001	590	<0.001	478	<0.001	243	<0.001
Slow-states R	536	<0.001	657	<0.001	541	<0.001	694	<0.001	514	<0.001
Slow-states Error	518	<0.001	559	<0.001	445	<0.001	427	<0.001	175	<0.001
Mixed-states R	572	<0.001	652	<0.001	506	<0.001	691	<0.001	483	<0.001
Mixed-states Error	543	<0.001	531	<0.001	422	<0.001	412	<0.001	174	<0.001

*Significance corresponds to a significant change in modulation of the error or correlation with ground truth (R) for different window lengths.*

### Two Node System: Sensitivity in Detecting Dynamic Connectivity for Different SNRs

**FIGURE 8:**
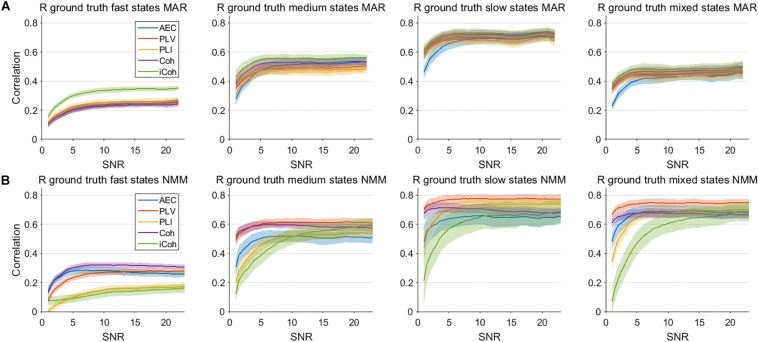
Correlation between connectivity estimates and ground truth for different levels of SNR for the two node MAR and NMM models. Panels **(A,B)** show the correlation between interpolated connectivity estimates and ground truth connectivity timecourse for different levels of SNR for the MAR and NMM model, respectively. Shaded areas correspond to the range of correlation values.

An important hurdle in the estimation of dynamic connectivity is the limited SNR of the data due to the inclusion only a relatively small number of samples. We therefore calculated the correlation of the connectivity estimates with the ground truth for various SNRs, and for both models (Figure 8). Results are only shown for the window length that matches the underlying state duration, i.e., the most optimal condition. For correlation with the ground truth the most important observation is that increases in SNR only lead to moderate increase of the correlation. Note that for all metrics, state durations and models, an increase in SNR eventually leads to a plateau where further increases in SNR hardly affects the correlation with the ground truth. The imaginary coherence seem to outperform the other metrics for fast state durations for the MAR model, which is the opposite for the NMM model. For the MAR model, for lower values of SNR the AEC seems to perform worse for slow states compared to the other metrics.

### Network Analysis: Detecting Temporal Fluctuations of Resting State Networks

**FIGURE 9:**
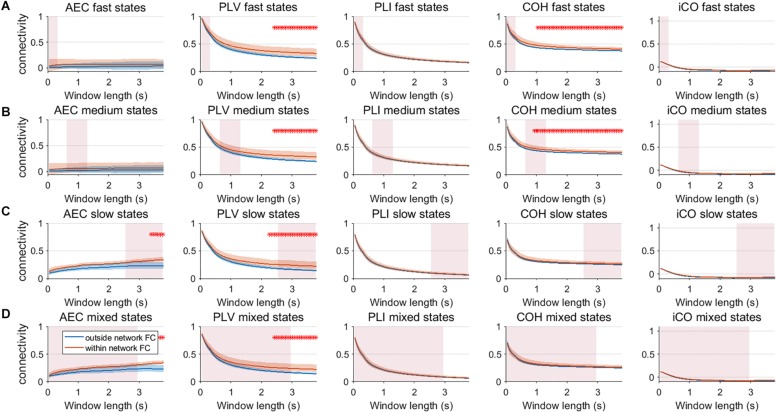
Within resting state network connectivity vs. outside resting state network connectivity (SNR = 3) based on the NMM model. Curves show mean within resting state subnetwork connectivity (red curves) and mean connectivity outside the resting state networks (blue curve) for different window lengths, state durations (duration of activity of the resting state subnetworks **(A–D)**) and connectivity metrics. Pink rectangles show the range of the underlying state durations. Shaded areas around the curves correspond to the range of values. A red cross in each panel corresponds to a significant difference in connectivity within vs. outside the resting state subnetworks (Mann–Whitney test *p* < 0.01) for the window length of interest.

We extended our two node analysis to a large scale network analysis. Figure 9 shows the results for neural mass simulations with an SNR = 3 and without linear mixing. For every mean resting state subnetwork duration of activity (fast – A, medium – B, slow – C, mixed states – D), we show functional connectivity within the active resting state subnetworks and functional connectivity outside of the resting state subnetworks. For the AEC, PLV and COH, we can observe that the estimate of connectivity within the resting state networks is higher than outside of these networks, especially for longer window lengths. PLI and iCO fail to show significant higher connectivity within resting state networks compared to outside network connectivity. Similarly as for the two node system, the curve of connectivity versus window length strongly depends on the selected window length rather than on the underlying duration of activity of the resting state subnetworks. Note that mostly there was a mismatch between the window length that showed significant differences and the underlying state durations, e.g., PLV and COH showed higher connectivity for resting state subnetworks compared to connectivity outside the subnetworks for longer window lengths than the underlying state durations (see Figure 9A). However, for AEC and PLV this also happens for longer window lengths that match the underlying dynamics of the slow states.

### Network Analysis: Detecting Temporal Fluctuations of Resting State Networks With Linear Mixing

**FIGURE 10:**
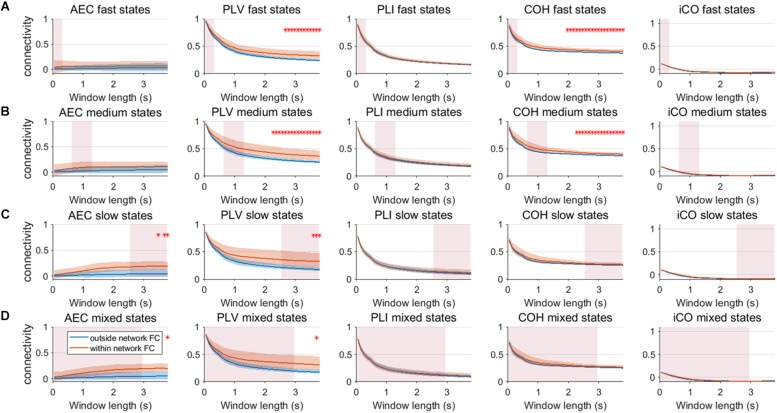
Within resting state network connectivity vs. outside resting state network connectivity (SNR = 5) based on the NMM model with linear mixing and symmetric leakage correction. Curves show mean within resting state subnetwork connectivity (red curves) and mean connectivity outside the resting state networks (blue curve) for different window lengths, state durations (duration of activity of the resting state subnetworks **(A–D)**) and connectivity metrics. Pink rectangles show the range of the underlying state durations. Shaded areas around the curves correspond to the range of values. A red cross in each panel corresponds to a significant difference in connectivity within vs. outside the resting state subnetworks (Mann–Whitney test *p* < 0.01) for the window length of interest.

We repeated the same analysis as in the previous section for a connected network of neural mass with linear mixing for different resting state network durations (fast – A, medium – B, slow – C, mixed states – D). Results for an SNR = 3 did not show any significant difference between connectivity within resting state networks and connectivity outside resting state networks (Supplementary Figure S9). Figure 10 shows the result for SNR = 5. Again, the curve for connectivity versus window is for all metrics very similar for different state durations, i.e., again the window length has a larger effect on estimations of connectivity than the underlying state durations. Within resting state connectivity is again higher for the AEC, PLV and COH, especially for longer window lengths. The window length for which there is significant difference between within resting state connectivity and connectivity outside the networks is on average longer than without linear mixing and symmetric leakage.

**FIGURE 11:**
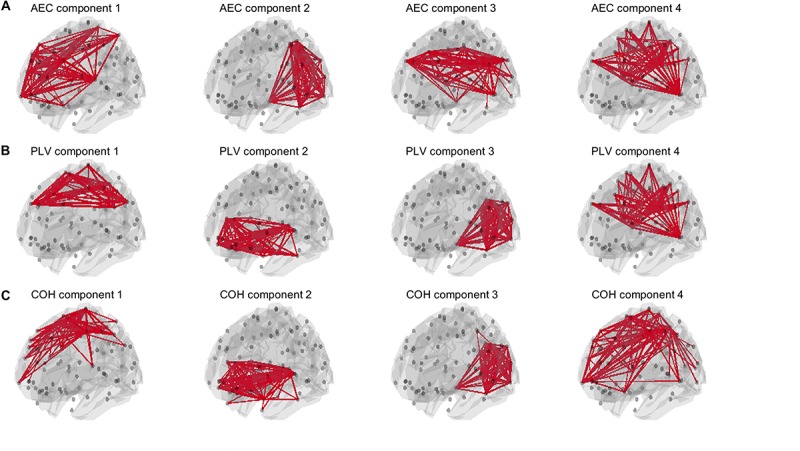
Estimated spatial patterns of time varying networks using non-negative tensor factorization. Results are shown for an SNR = 5 in a NMM model with linear mixing and symmetric leakage correction and for a window length of 4 s with slow underlying state durations. The upper row **(A)** shows estimated networks for the AEC (amplitude envelope correlation). The second row **(B)** shows estimated networks for the PLV (phase locking value), while the third row **(C)** shows estimated networks for the COH (Coherence). The upper 3% of the connections within each component is illustrated. Note that for all metrics some of the *a priori* defined networks could be retrieved. *A priori* defined networks were the default mode network (DMN), the sensorimotor network (SMN), the frontoparietal networks (FPN) and the visual network (see Figure 2).

For the AEC, PLV and COH, we tested for window lengths of 4 s whether we could retrieve the spatial patterns of the a-prior defined resting state networks. i.e., the DMN, the SMN, the FPN and the visual network (Figure 2). Figure 11 shows the spatial patterns of the estimated time varying networks for the slow states. Components estimated from AEC resembled three *a priori* defined networks, while components extracted from the PLV resembled two apparent resting state networks, and for COH three clearly recognizable networks could be obtained. For example, for the AEC, non-negative tensor factorization retrieved a FPN with predominantly connections on the right, the visual network, default mode network, but no clear sensorimotor network. For the PLV we could observe a sensorimotor network and visual network, and a network reminiscent of the default mode network. Lastly, for the COH we could observe a clear visual network and two networks that had some spatial characteristics of the sensorimotor and FPN. To emphasize our previous analysis (shown in Figure 10), we could not identify clear-cut networks for medium states and window lengths of 1 s for all the metrics, though the visual network was an exception (see Supplementary Figure S10). As was also hinted in Figure 10, for medium states, extraction of resting state networks improved for using longer windows (see Supplementary Figure S11), though networks were not as clearly recognizable as for slow states.

## Discussion

Despite advances in the field of dynamic connectivity, fixed sliding window approaches in conjunction with conventional metrics are still widely used to identify dynamic connectivity. Given the lack of ground truth in empirical MEG data, we used simulations based on parameterized MAR and NMM models informed by predefined timecourses of connectivity. We performed a step by step analysis in a two node system and in a large scale network. Two node analysis revealed that SNR should be sufficiently large in order to distinguish static connectivity from dynamic connectivity. Especially excursions from the median was the most sensitive measure to distinguish dynamic connectivity from static connectivity. All connectivity metrics, performed well to detect fluctuations in slow dynamic states and to some extent medium dynamic states. However, the identification of fast underlying ground truth states (mean state duration 125ms) was poor for all metrics. An increase in SNR resulted in only a moderate increase in identification of the ground truth connectivity timecourses. Network analysis indeed underscored that resting state networks could only be retrieved for sufficient levels of SNR and long state durations.

Variability has often been used as the outcome measure to quantify the dynamics of connectivity. In the current work variability was quantified by the standard deviation of connectivity across estimates from all sliding windows (with a constant width). Here, we show that variability unequal to zero in itself does not necessarily imply evidence for a dynamic underlying system (Figure 3). For both static and dynamic connectivity there was high variability for short window lengths, indicating that high variability can merely be an artifact of the window length. Also note that high variability for short states (Figure 3A) could co-occur with poor identification of the underlying connectivity timecourse (Figures 7C,D). Especially, for low SNR (SNR < 3), for fast and medium states variability could hardly distinguish genuine dynamic connectivity from static connectivity. Another hurdle with the use of the standard deviation of connectivity is that the connectivity distributions are mostly non-Gaussian, as evidenced by non-zero skewness. The skewness of the distribution was a more sensitive statistic to detect differences in static vs. dynamic connectivity, especially for the phase based metrics, though the window lengths for which this occurred did not necessarily match the underlying state durations. The kurtosis was found to be less useful in disentangling dynamic from static connectivity, while excursions from the median was sensitive for detecting genuine fluctuations in connectivity, especially for the window widths that match the underlying state duration. However, note that for both skewness and excursion, their magnitude is highly influenced by the width of the window.

Unlike in empirical data, we could test the performance of the metrics with an underlying ground truth. Results were fairly similar for the MAR and NMM simulations. All conventional metrics performed poorly when the ground truth contained fast states, as their correlation with the underlying ground truth timecourse was low. In other words, metrics are unable to quantify very brief states in a fixed sliding window approach. This therefore limits the use of fixed sliding window approaches for the detection of fast states. Our results showed that sliding window approaches are safe to use if the underlying states are at least of medium duration. Even an increase in SNR could not significantly improve the performance of the metrics for the fast states, implying that the low performance is not only related to SNR but also to the properties of the metrics. Recent studies have shown that in empirical resting state data fluctuations in amplitude and phase coupling can be well-described in a range from a few hundred milliseconds to seconds (Baker et al., 2014; Tewarie et al., 2018; Vidaurre et al., 2018). We mimicked this by the mixed state condition, which actually also show very moderate correlation for different metrics with the underlying ground truth, indicating that there is high uncertainty in detection of the underlying connectivity. Finally, the correlation with the ground truth was usually maximal for window lengths that more or less matched the state durations (except for longer state durations). A mismatch between window length and underlying state duration also led to a drop in correlation with the ground truth. This emphasizes the problem of an arbitrary window length for the estimation of dynamic connectivity with unknown, and most likely varying, state durations.

Another important observation is that one would beforehand expect that phase-based metrics would perform better for faster states, whereas the amplitude envelope correlation would perform better for slower states, since the amplitude envelopes modulates on slower timescales than the phases. This was especially obvious for the network simulations, where PLV and coherence were sensitive in detecting medium state durations, while AEC was only able to detect network connectivity for slow and mixed state durations. However, at the same time, this notion requires caution since performance of metrics seem to depend on the type of simulation (MAR vs. NMM). For example, in the MAR model, there is explicit parameterization of phase locking, which would favor estimates of phase locking over amplitude-amplitude coupling. Potentially, differences in MAR and NMM results could also be explained by differences in the intrinsic frequencies of the oscillations, i.e., beta band oscillations in MAR vs. alpha band oscillations in NMM. This may also explain the observed difference in performance of imaginary coherence for NMM vs. MAR simulations. Also note, that for most simulations, performance of metrics nearly converge for sufficient SNR or longer window lengths (see Figures 7,  8).

Our two node analysis was extended to a more realistic large scale network scenario. For simulations without linear mixing and leakage correction, metrics that were inherently sensitive for leakage, AEC, PLV and coherence were sensitive in detecting genuine fluctuations in connectivity. Coherence was even sensitive to detect within resting state network connectivity for medium state durations for windows that matched these state durations. However, this was also the case for longer window lengths that did not match the underlying state duration. Thus this indicates that significant higher connectivity, for a resting state network for a specific window length, does not necessarily imply that the underlying duration of temporal varying connectivity has the same temporal scale as the window width. Especially slow states were detectable by the AEC, PLV and COH for longer window widths. For the scenario with linear mixing and symmetric leakage correction, results showed that to extract meaningful resting state networks, we usually required longer windows, especially for the AEC. This finding is in line with empirical work, where meaningful time varying networks for AEC could be extracted during a working memory task for longer window widths (O’Neill et al., 2017a).

Some methodological issues warrant further discussion. A limitation of the current work is that our simulations are not necessarily a direct representation of electrophysiological data. However, a methodological issue with empirical data is its lack of ground truth. Connectivity analysis with empirical data would require the use of surrogate data (Dimitriadis et al., 2018). Previous work has illustrated that the choice and selection of surrogate methods is not trivial (O’Neill et al., 2017b) and conclusions regarding non-stationarity of connectivity based on surrogate data can be highly biased by the selection of the method itself. Extensive analysis of various surrogate methods in the context of dynamic connectivity is beyond the scope of this paper, but should surely be explored in future work (Dimitriadis et al., 2018). Another limitation is that other metrics of functional connectivity such as mutual information (Palus, 1997) or measures that characterize generalized synchronization, such the synchronization likelihood (Stam and Van Dijk, 2002), as well as metrics that estimate directed connectivity (Nolte et al., 2010; Lobier et al., 2014) were not included in our analysis. Here, we restricted our analysis to frequently used (and computationally inexpensive) phase- and amplitude- based functional connectivity metrics. In addition, ideally the effects of co-registration, lead field inaccuracies, and effects of inverse operator would also be simulated (Hincapié et al., 2017; Chella et al., 2019). However, these errors lead to reduced amplitude of the reconstructed signal. As a surrogate, and in order to reduce the number of simulations, these effects can be observed by examining the effects of SNR on our results. Lastly, network simulations were only performed for the NMM, since to boost neurobiological realism, we could implement distance dependent conduction delays. Implementing these distance dependent delays is not trivial in a linear MAR model, and therefore the MAR model was not used for this purpose.

In conclusion, we have demonstrated the strengths and limitations of metrics based on the two intrinsic modes of coupling (amplitude and phase) with regards to the detection of genuine fluctuations in functional connectivity. Fixed sliding window approaches have difficulty in detecting brief states, even when using short window lengths. Increasing SNR does not mitigate this sufficiently, especially for large scale networks. We therefore recommend the use of longer window lengths (at least 3–4 s) to estimate fluctuations in functional connectivity of resting state networks. Our simulations showed that PLV, AEC and coherence outperform imaginary coherence and PLI, which might advocate for the use of the former three metrics for the estimation of dynamic functional connectivity. Furthermore, an often used metric to quantify dynamic FC, variability, also comes with difficulties: high variability could co-occur with low correlation with the ground truth and can be an artifact of the used window length. Given the non-Gaussianity of most connectivity distributions, skewness may be a more appropriate metric to quantify genuine fluctuations in connectivity and in addition, excursions from the median could also be used in this context. However, again the magnitude of these metrics can be merely an artifact of the selected window length, and these metrics provide only meaningful information if they are tested against a null-hypothesis of static connectivity. Caution is therefore warranted when using these outcome measures in empirical data.

## Data Availability

All datasets generated for this study are included in the manuscript and/or the Supplementary Files.

## Author Contributions

LL, AQ, MW, MB, and GO designed the study. AQ and LL wrote the code. LL, PT, and AQ analyzed the data. LL, AQ, GO, MW, AH, and PT wrote and edited the manuscript. All authors contributed to interpretation of results.

## Conflict of Interest Statement

The authors declare that the research was conducted in the absence of any commercial or financial relationships that could be construed as a potential conflict of interest.
