# Supplementary material for: How Sensitive Are Conventional MEG Functional Connectivity Metrics With Sliding Windows to Detect Genuine Fluctuations in Dynamic Functional Connectivity?
Source: Front Neurosci. 2019 Aug 2;13:797. doi: 10.3389/fnins.2019.00797 (PMC6688728; doi:10.3389/fnins.2019.00797)
Supplement: Supplementary file 1 [file Data_Sheet_1.docx]

***Figure S1: Two node MAR model summary statistics in connectivity in a static vs medium dynamic underlying system (SNR = 1).*** *Panel A shows the standard deviation of connectivity across the number of windows for the static and dynamic underlying system (pink rectangles correspond to the underlying ground-truth of the range of the state durations). Panel B shows the skewness of the connectivity distributions. Panel C shows the kurtosis of the connectivity distributions and panel D the excursions from the median. Error bars correspond to the range across realisations/iterations. A red cross corresponds to a significant difference in distribution between a summary statistic for static and dynamic connectivity (Mann-Whitney test p < 0.01) for the window length of interest.*

***Figure S2: Two node MAR model summary statistics in connectivity in a static vs mixed dynamic underlying system (SNR = 1).*** *Panel A shows the standard deviation of connectivity across the number of windows for the static and dynamic underlying system (pink rectangles correspond to the underlying ground-truth of the range of the state durations). Panel B shows the skewness of the connectivity distributions. Panel C shows the kurtosis of the connectivity distributions and panel D the excursions from the median. Error bars correspond to the range across realisations/iterations. A red cross corresponds to a significant difference in distribution between a summary statistic for static and dynamic connectivity (Mann-Whitney test p < 0.01) for the window length of interest.*

***Figure S3: Two node NMM model summary statistics in connectivity in a static vs medium dynamic underlying system (SNR = 1).*** *Panel A shows the standard deviation of connectivity across the number of windows for the static and dynamic underlying system (pink rectangles correspond to the underlying ground-truth of the range of the state durations). Panel B shows the skewness of the connectivity distributions. Panel C shows the kurtosis of the connectivity distributions and panel D the excursions from the median. Error bars correspond to the range across realisations/iterations. A red cross corresponds to a significant difference in distribution between a summary statistic for static and dynamic connectivity (Mann-Whitney test p < 0.01) for the window length of interest.*

***Figure S4: Two node NMM model summary statistics in connectivity in a static vs slow dynamic underlying system (SNR = 1).*** *Panel A shows the standard deviation of connectivity across the number of windows for the static and dynamic underlying system (pink rectangles correspond to the underlying ground-truth of the range of the state durations). Panel B shows the skewness of the connectivity distributions. Panel C shows the kurtosis of the connectivity distributions and panel D the excursions from the median. Error bars correspond to the range across realisations/iterations. A red cross corresponds to a significant difference in distribution between a summary statistic for static and dynamic connectivity (Mann-Whitney test p < 0.01) for the window length of interest.*

***Figure S5: Two node NMM model summary statistics in connectivity in a static vs mixed dynamic underlying system (SNR = 1).*** *Panel A shows the standard deviation of connectivity across the number of windows for the static and dynamic underlying system (pink rectangles correspond to the underlying ground-truth of the range of the state durations). Panel B shows the skewness of the connectivity distributions. Panel C shows the kurtosis of the connectivity distributions and panel D the excursions from the median. Error bars correspond to the range across realisations/iterations. A red cross corresponds to a significant difference in distribution between a summary statistic for static and dynamic connectivity (Mann-Whitney test p < 0.01) for the window length of interest.*

***Figure S6: Two node MAR model summary statistics in connectivity in a static vs medium dynamic underlying system (SNR = 3).*** *Panel A shows the standard deviation of connectivity across the number of windows for the static and dynamic underlying system (pink rectangles correspond to the underlying ground-truth of the range of the state durations). Panel B shows the skewness of the connectivity distributions. Panel C shows the kurtosis of the connectivity distributions and panel D the excursions from the median. Error bars correspond to the range across realisations/iterations. A red cross corresponds to a significant difference in distribution between a summary statistic for static and dynamic connectivity (Mann-Whitney test p < 0.01) for the window length of interest.*

***Figure S7: Two node MAR model summary statistics in connectivity in a static vs slow dynamic underlying system (SNR = 3).*** *Panel A shows the standard deviation of connectivity across the number of windows for the static and dynamic underlying system (pink rectangles correspond to the underlying ground-truth of the range of the state durations). Panel B shows the skewness of the connectivity distributions. Panel C shows the kurtosis of the connectivity distributions and panel D the excursions from the median. Error bars correspond to the range across realisations/iterations. A red cross corresponds to a significant difference in distribution between a summary statistic for static and dynamic connectivity (Mann-Whitney test p < 0.01) for the window length of interest.*

***Figure S8: Two node MAR model summary statistics in connectivity in a static vs mixed dynamic underlying system (SNR = 3).*** *Panel A shows the standard deviation of connectivity across the number of windows for the static and dynamic underlying system (pink rectangles correspond to the underlying ground-truth of the range of the state durations). Panel B shows the skewness of the connectivity distributions. Panel C shows the kurtosis of the connectivity distributions and panel D the excursions from the median. Error bars correspond to the range across realisations/iterations. A red cross corresponds to a significant difference in distribution between a summary statistic for static and dynamic connectivity (Mann-Whitney test p < 0.01) for the window length of interest.*

***Figure S9: within resting state network connectivity vs outside resting state network connectivity (SNR = 3) based on the NMM model with linear mixing and symmetric leakage correction.*** *Curves show mean within resting state network connectivity (red curves) and mean connectivity outside the resting state networks for different window lengths, state durations (duration of activity of the resting state networks) and metrics. Shaded areas correspond to the range of values. A red cross in each panel corresponds to a significant difference in connectivity within vs outside the resting state networks (Mann-Whitney test p < 0.01) for the window length of interest.*

***Figure S10: Estimated spatial patterns of time varying networks using non-negative tensor factorisation.*** *Results are shown for an SNR = 5 in a NMM model with linear mixing and symmetric leakage correction and for a window length of one second with medium underlying state durations. The upper row (A) shows estimated networks for the AEC (amplitude envelope correlation). The second row (B) shows estimated networks for the PLV (phase locking value), while the third row shows estimated networks for the COH (Coherence). The upper 3% of the connections within each component is illustrated. Note that for all metrics some of the a-priori defined networks could be retrieved. A-priori defined networks were the default mode network (DMN), the sensorimotor network (SMN), the frontoparietal networks (FPN) and the visual network (see Figure 2).*

***Figure S11: Estimated spatial patterns of time varying networks using non-negative tensor factorisation.*** *Results are shown for an SNR = 5 in a NMM model with linear mixing and symmetric leakage correction and for a window length of four seconds with medium underlying state durations. The upper row (A) shows estimated networks for the AEC (amplitude envelope correlation). The second row (B) shows estimated networks for the PLV (phase locking value), while the third row shows estimated networks for the COH (Coherence). The upper 3% of the connections within each component is illustrated. Note that for all metrics some of the a-priori defined networks could be retrieved. A-priori defined networks were the default mode network (DMN), the sensorimotor network (SMN), the frontoparietal networks (FPN) and the visual network (see Figure 2).*
