# Supplementary figures and images for: How Sensitive Are Conventional MEG Functional Connectivity Metrics With Sliding Windows to Detect Genuine Fluctuations in Dynamic Functional Connectivity?
Source: Front Neurosci. 2019 Aug 2;13:797. doi: 10.3389/fnins.2019.00797 (PMC6688728; doi:10.3389/fnins.2019.00797)

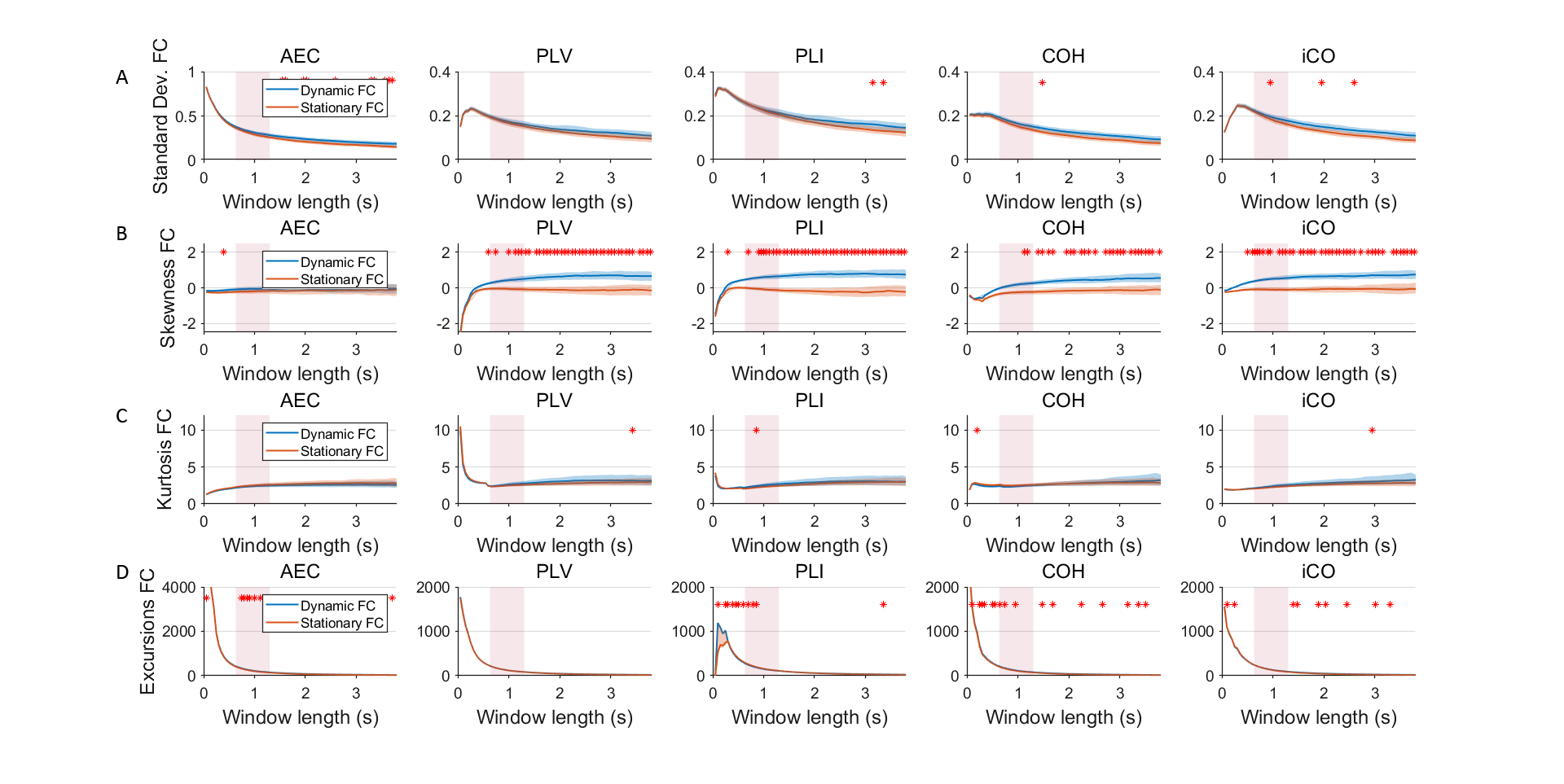

Supplement: Supplementary file 2 [file Image_1.TIF]

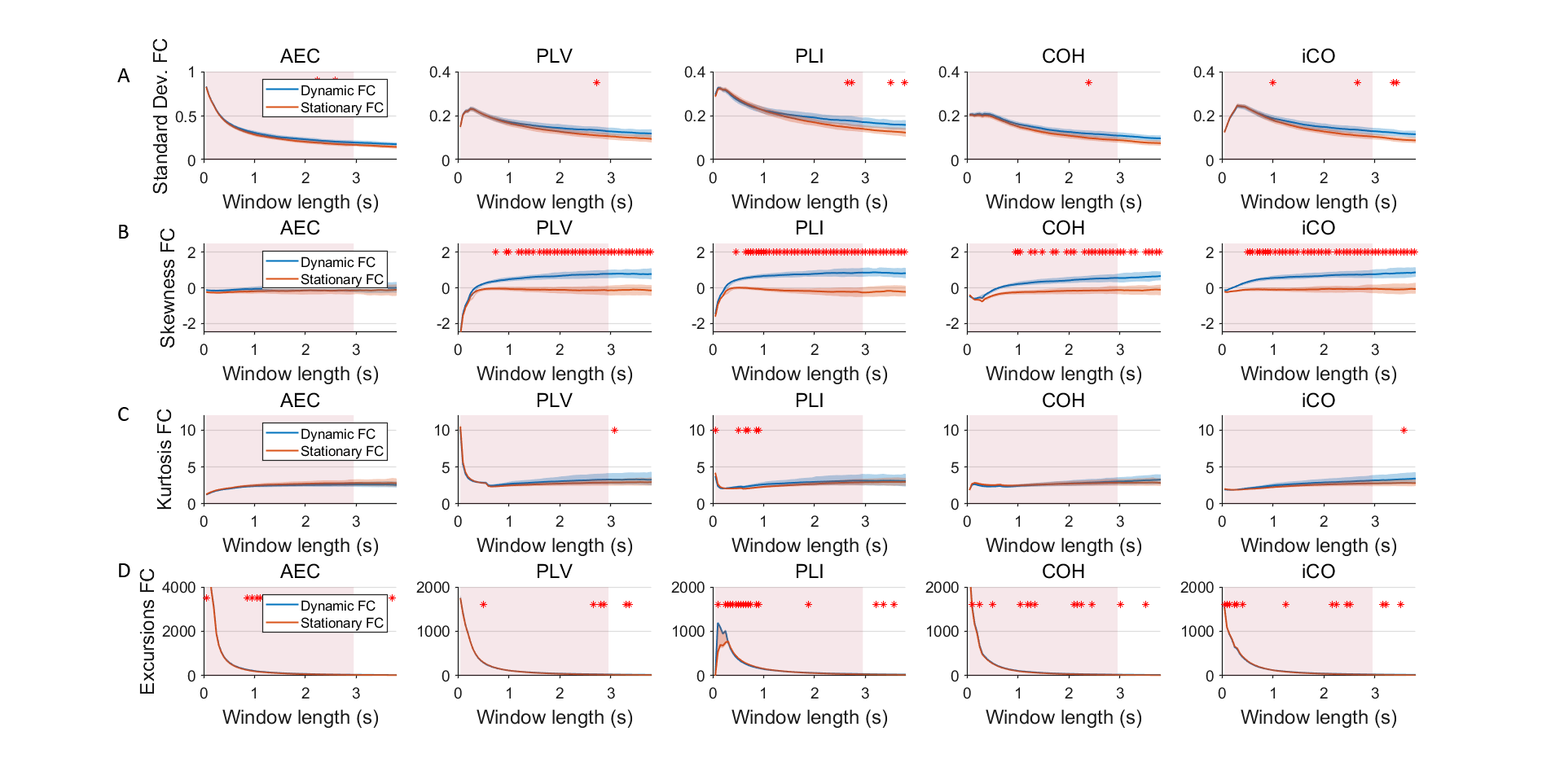

Supplement: Supplementary file 3 [file Image_2.TIF]

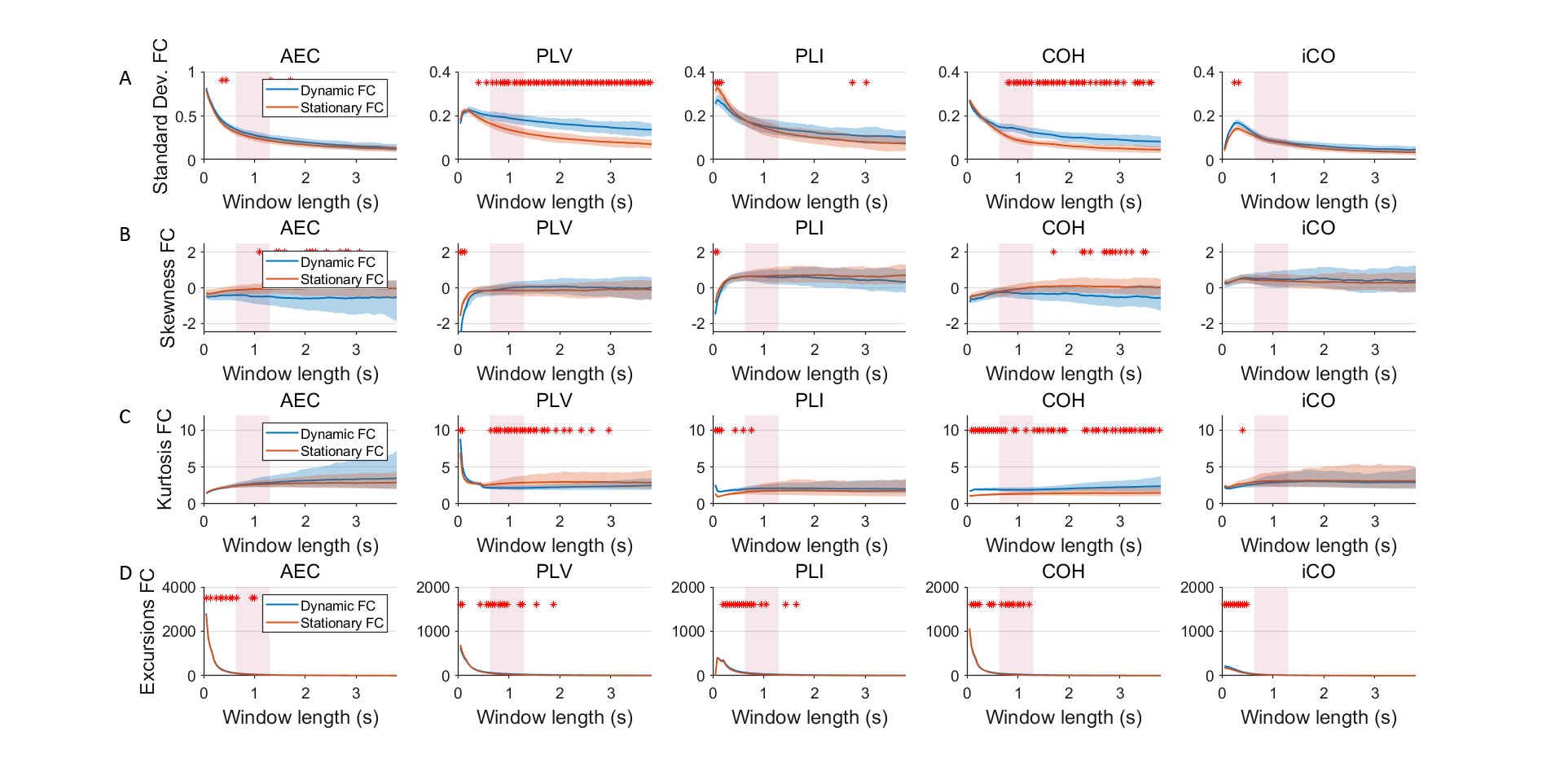

Supplement: Supplementary file 4 [file Image_3.TIF]

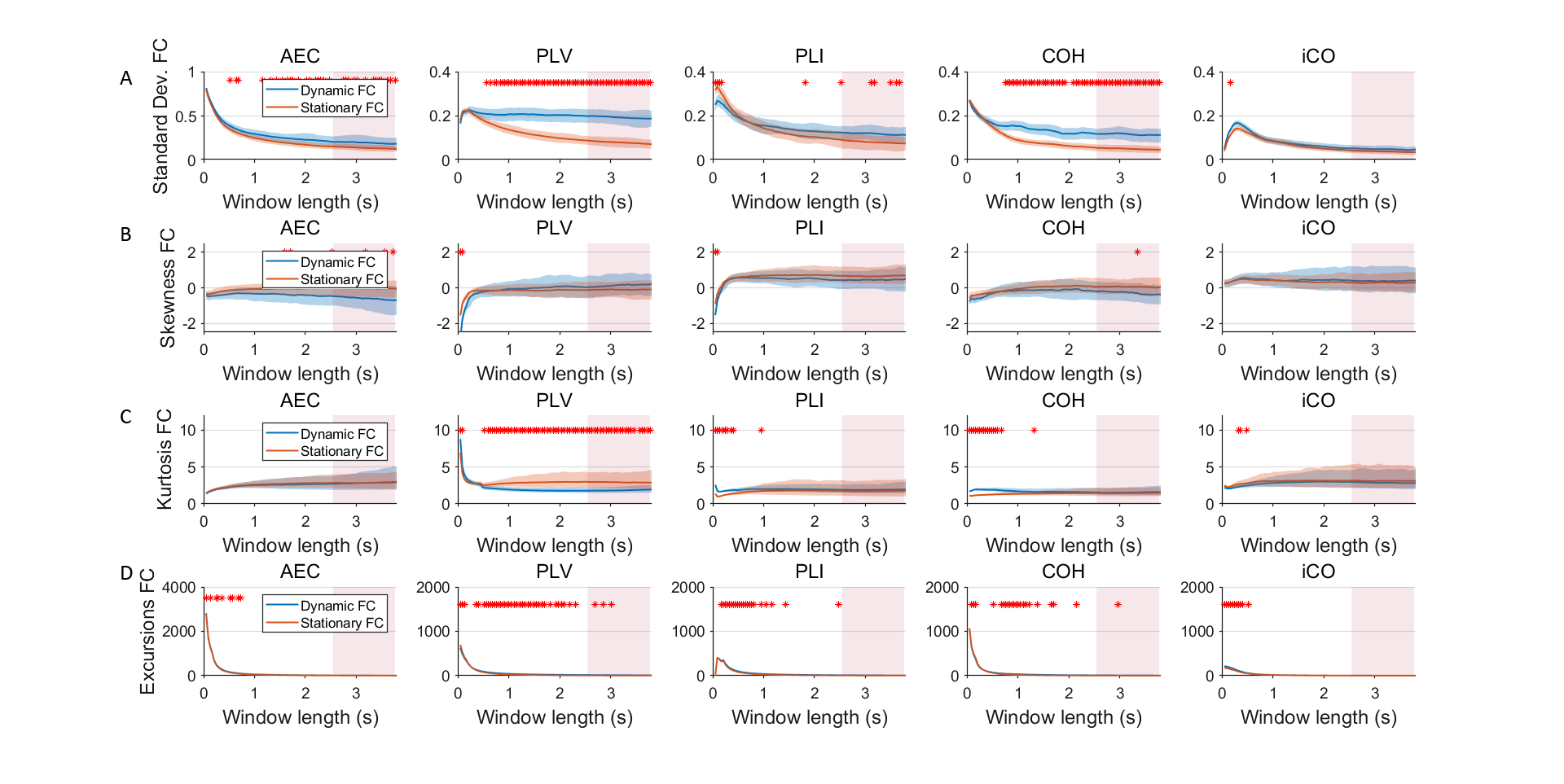

Supplement: Supplementary file 5 [file Image_4.TIF]

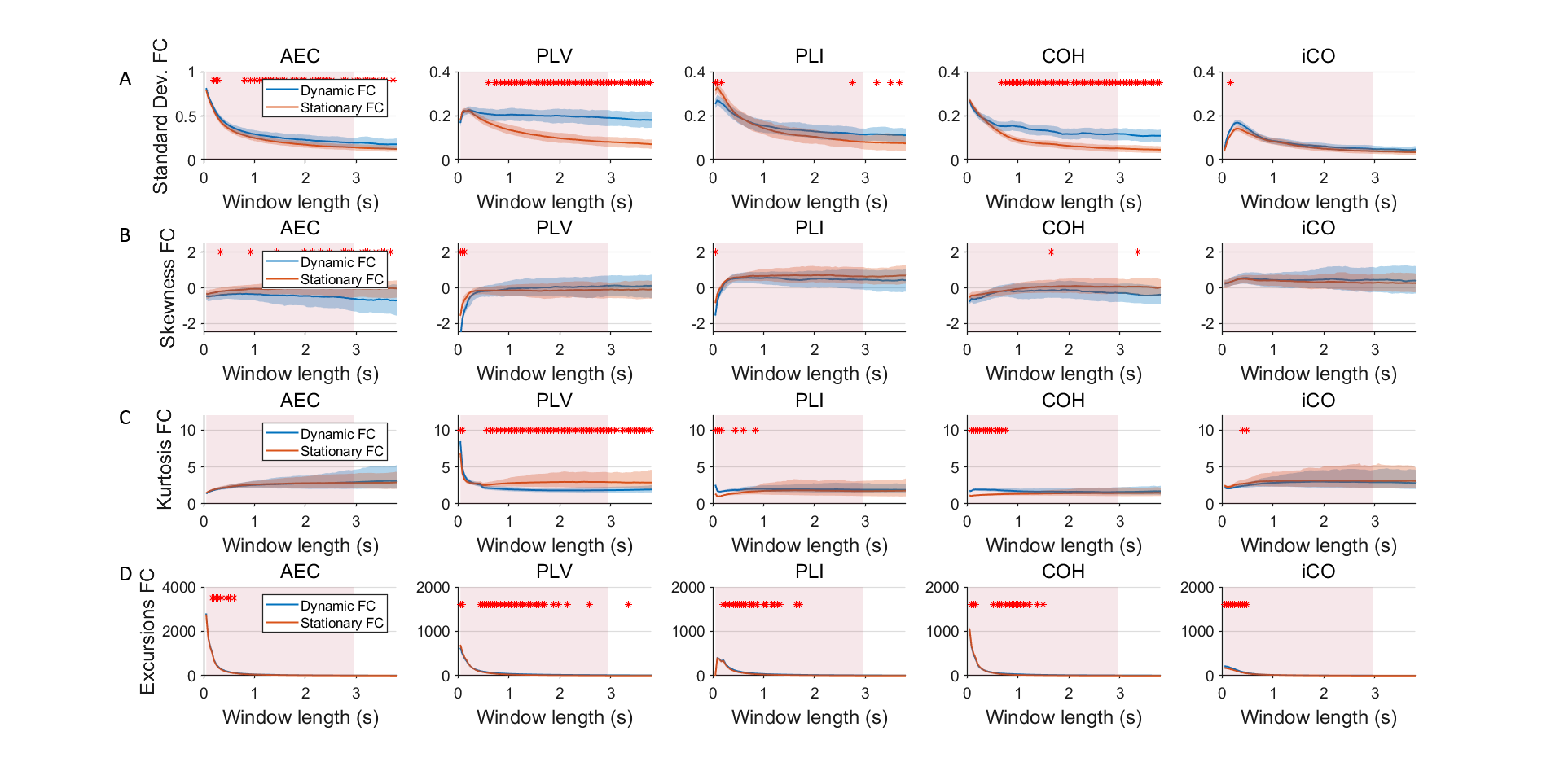

Supplement: Supplementary file 6 [file Image_5.TIF]

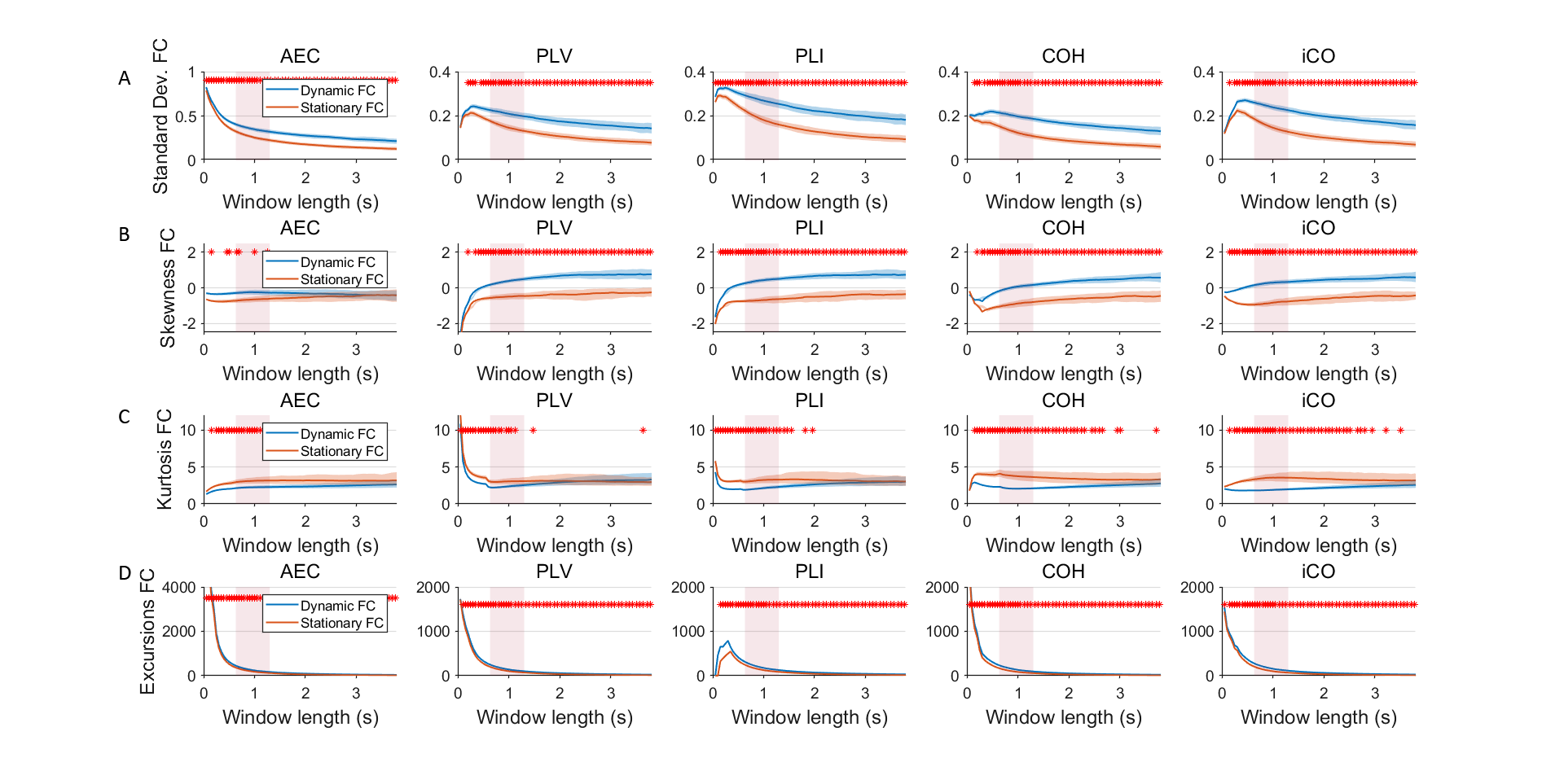

Supplement: Supplementary file 7 [file Image_6.TIF]

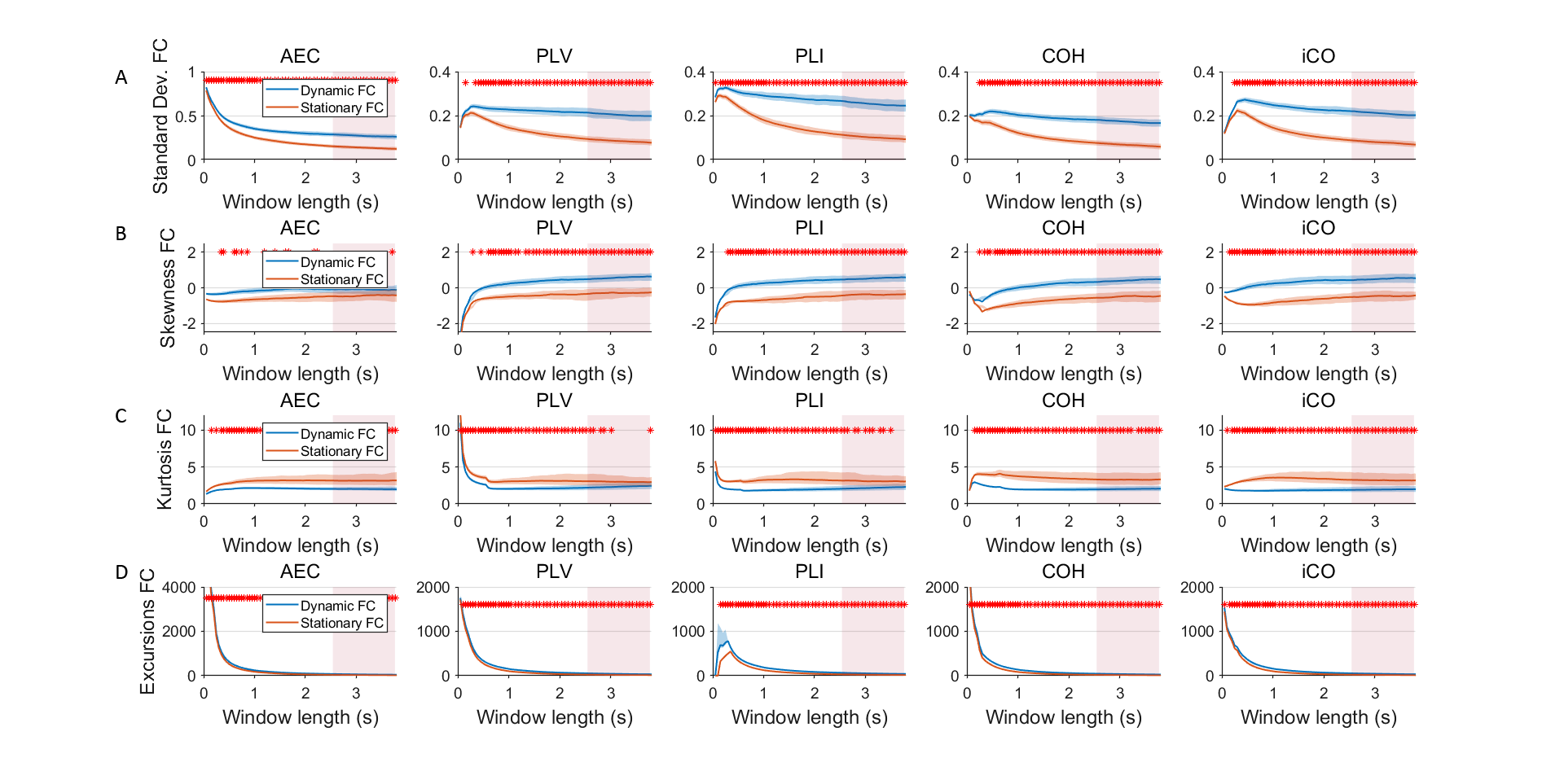

Supplement: Supplementary file 8 [file Image_7.TIF]

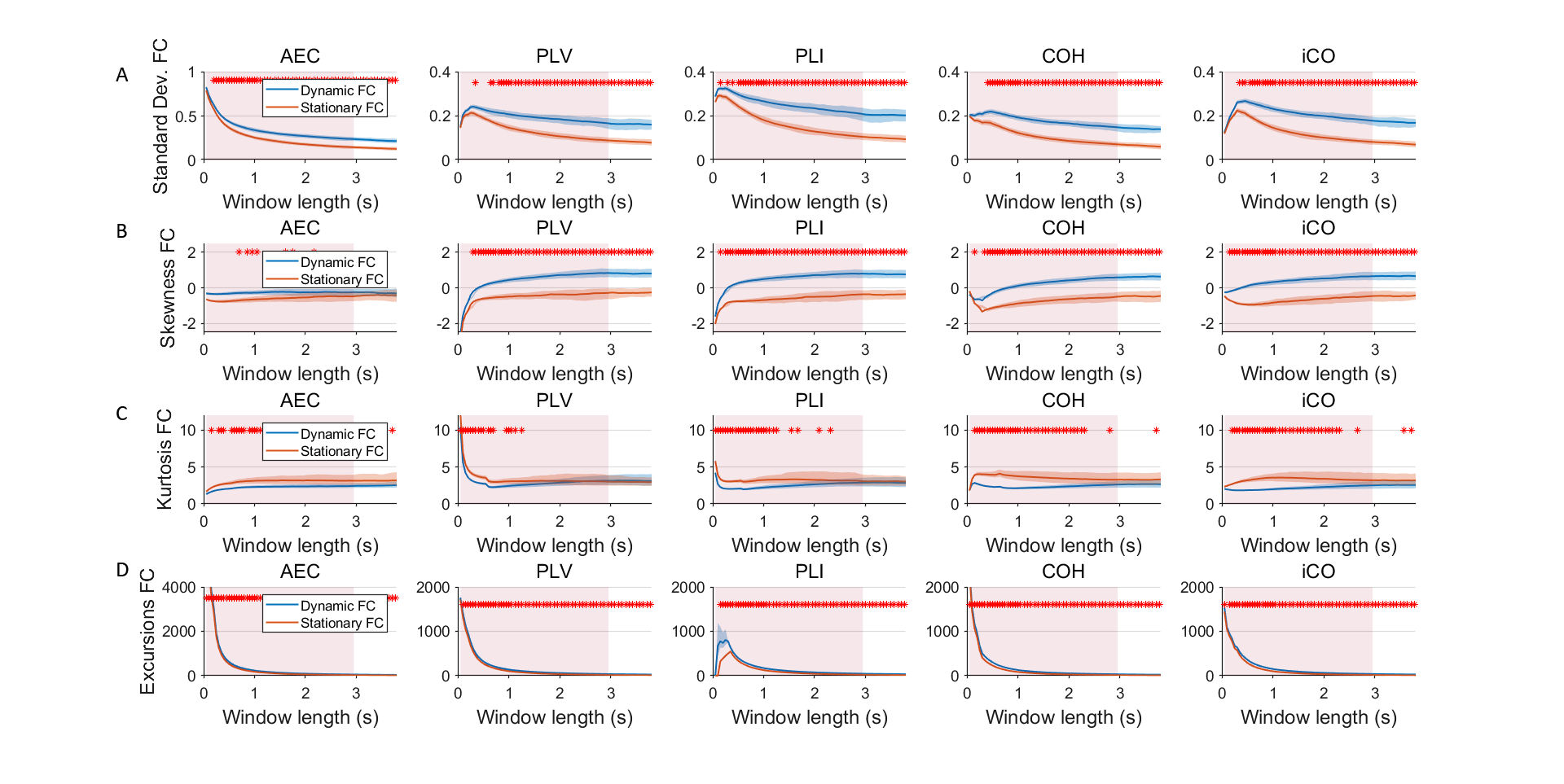

Supplement: Supplementary file 9 [file Image_8.TIF]

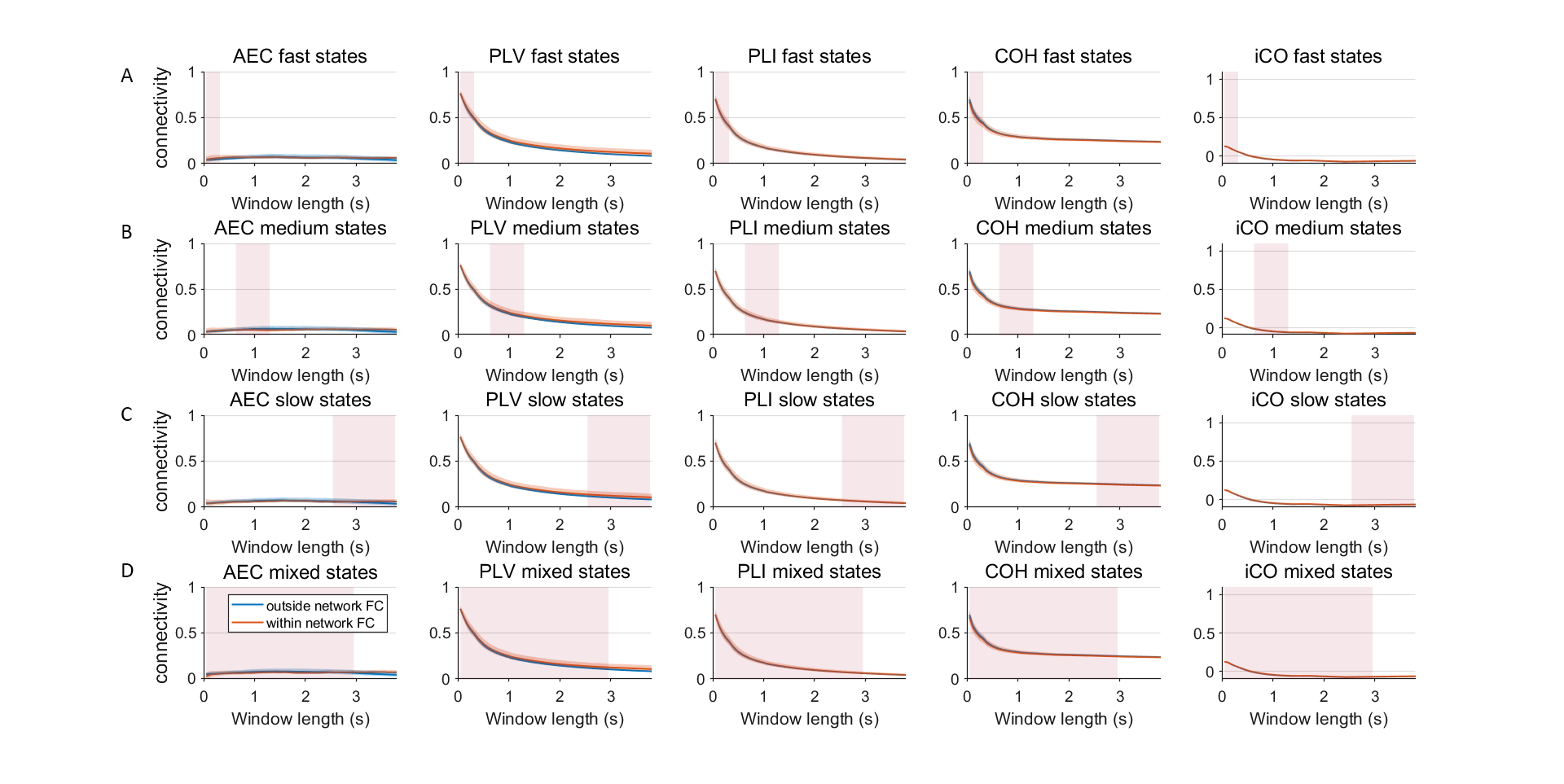

Supplement: Supplementary file 10 [file Image_9.TIF]

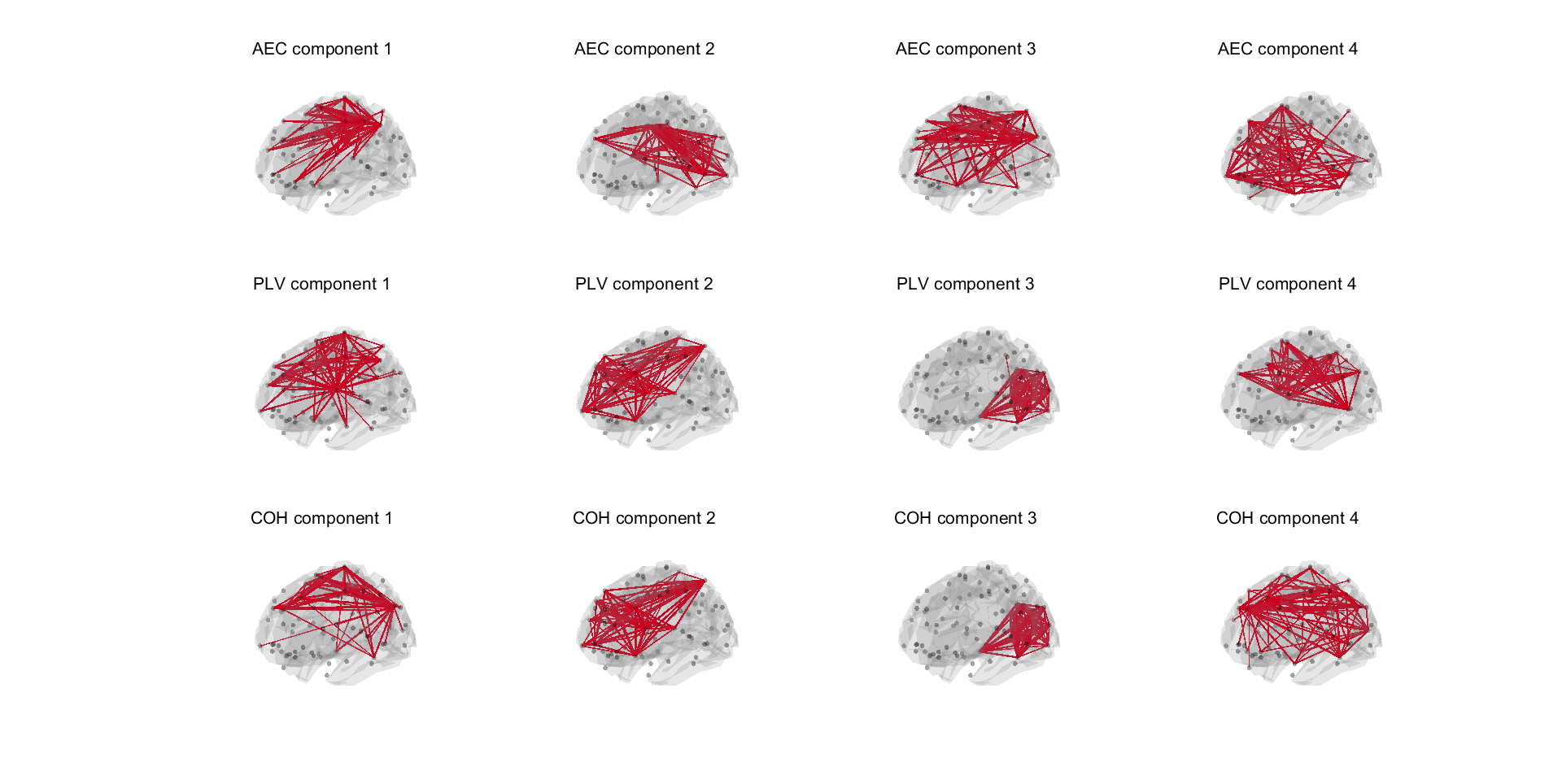

Supplement: Supplementary file 11 [file Image_10.TIF]

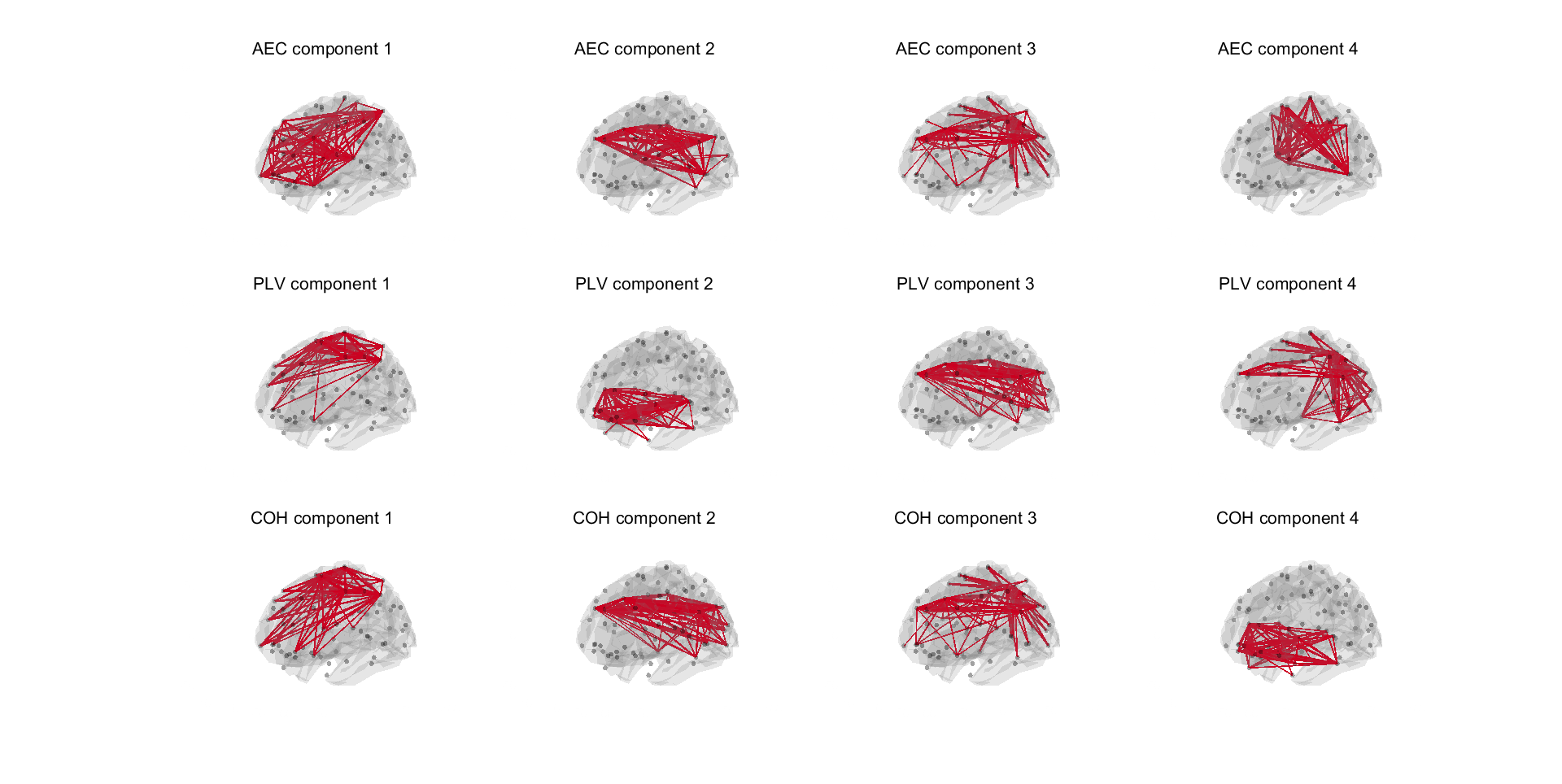

Supplement: Supplementary file 12 [file Image_11.TIF]
